# Physics-Driven Computational Multispectral Imaging for Accurate Color Measurement

**DOI:** 10.3390/s25175443

**Published:** 2025-09-02

**Authors:** Haoyu Yi, Mingwei Zhou, Hao Xie, Bingshan Chen, Yaqi Wang, Fei Liu, Jiefei Shen, Junfei Shen

**Affiliations:** 1 College of Electronics and Information Engineering, Sichuan University, Chengdu 610065, China; yhyyyds315@gmail.com (H.Y.); hxie@stu.scu.edu.cn (H.X.); j65822304@gmail.com (B.C.); yaqiw.ece@utexas.edu (Y.W.); 2 Mindray Bio-Medical Electronics Co., Ltd., Shenzhen 518132, China; 13096300597@163.com; 3 Department of Prosthodontics, West China Hospital of Stomatology, Sichuan University, Chengdu 610041, China; liufei.hxkq@scu.edu.cn (F.L.); shenjiefei@scu.edu.cn (J.S.)

**Keywords:** color measurement, spectral reflectance, deep learning, physically informed network, hyperspectral imaging

## Abstract

Accurate color measurement is crucial for ensuring reliable sensing performance in vision-based applications. However, existing color measurement methods suffer from illumination variability, operational complexity, and perceptual subjectivity. In this study, dental color measurement, with its strict perceptual and spectral fidelity demands, is adopted to validate the proposed method. Using self-made resin-permeated ceramic teeth, this study proposes a deep-learned end-to-end spectral reflectance prediction framework to achieve snapshot teeth spectral reflectance from RGB images under complex light sources in the fundamental spectral domain through the construction of a physically interpretable network that enables physically informed feature fusion. A dual-attention modular-information fusion neural network is developed to recover the spectral reflectance directly from the RGB image for natural teeth and ceramics across multiple scenarios. A dataset containing 4000 RGB–hyperspectral image pairs is built from a self-designed optical system with complex illumination conditions. Results confirm that the proposed framework demonstrates effective performance in predicting teeth spectral reflectance with an MSE of 0.0024 and an SSIM of 0.8724. This method achieves high-accuracy color measurement while avoiding the color mismatch caused by metamerism, which empowers various advanced applications including optical property characterization, 3D surface reconstruction, and computer-aided restorative design.

## 1. Introduction

As of 2022, approximately 3.5 billion people worldwide suffer from oral diseases, with untreated dental caries affecting around 2.5 billion individuals, highlighting the critical need for dental restoration [1]. The color of teeth serves as a significant determinant of individuals’ satisfaction with their dental and facial aesthetics [2], which plays a critical role in shaping social perceptions of personal attributes [3]. The color and appearance of teeth constitute a complex phenomenon influenced by multiple factors, including lighting conditions, translucency, opacity, light scattering, surface gloss, and human visual perception [4]. Therefore, achieving accurate and efficient dental color measurement represents a critical step in the process of dental restoration. Considering differences in the dimensionality and completeness of color data acquisition, most can be divided into two types: RGB analysis and spectral analysis.

**RGB analysis**. RGB analysis refers to the process of acquiring and interpreting color information based on the red, green, and blue components of an image. In dental color measurement, it involves assessing tooth color either visually by the human eye or through RGB color data captured by instruments. From an instrumentation perspective, RGB analysis methods can be broadly categorized into four types: visual assessment, colorimeters, digital cameras, and intraoral scanners (IOS).

(1) Visual analysis. This method is regarded as the gold standard in clinical application, relying on the subjective judgment of the dentist or technician, who compares the color of the patient’s natural teeth to a shade guide (a set of standardized color samples) to select the closest match. However, due to the subjectivity of human visual analysis and the variations in lighting conditions, visual determination of shade selection has been found to be subjective and inconsistent [5]. Historically, visual shade assessment has been inherently constrained by several factors, including metamerism, the observer’s age, visual fatigue, and the influence of mood or medication. For instance, color vision deteriorates with age [6], compromising the consistency of shade-based diagnosis. Metamerism is the matching of apparent color of objects with lights that have different spectral power distributions [7], which indicates the same color under one lighting condition may appear different under other conditions. Therefore, the ability to communicate the degree and nature of these differences is lacking [8], highlighting the pressing need for more objective, reproducible, and technology-assisted approaches in clinical shade selection.
(2) Colorimeters. Colorimeters measure color tristimulus values from light reflectance of a specimen after the light source pass through a series of filters [9]. When conducting color measurement, the intensity of electromagnetic radiation within the visible spectrum wavelengths of an object or solution after it has been transmitted or reflected will be measured. It can offer potential objective and quantitative assessment of tooth color, independent of the examiner’s experience and environmental conditions [10]. Repeatability may be compromised due to filter aging, and metamerism of the object can pose a challenge to measurement accuracy [11]. Moreover, if the angle or position of measurement is incorrect or deviates, it can lead to inconsistent results, especially for teeth that have irregular shapes or complex surface morphology. Such variations can introduce errors in color measurement.
(3) Digital cameras. The usage of digital cameras has been widely adopted in the fields of dentistry [12]. Shade matching using the digital images can minimize the gap of color communication between dentists and technicians [13]. The process of digital photographic color-matching emphasizes the importance of environmental lighting control, color calibration, and software analysis. However, color consistency can be influenced by various factors during image acquisition, including ambient lighting, tooth hydration or dehydration, camera settings, shade-taking protocols, use of cross-polarization, image file formats, color balance adjustments via shade analysis software, and calibration of laboratory monitors [14]. For example, unreliable illumination and unfixed distances confound the photographic outcome. In other words, variations in dental color measurement can result from differences in the light source’s brightness, hue, and proximity to the teeth.
(4) Intraoral scanners (IOSs). An IOS (intraoral scanner) is a medical device consisting of a handheld camera (hardware), a computer, and software [15]. Although intraoral scanners have been initially used for digital impression, a tool for dental shade measurement has been added to some scanners [14]. For intraoral scanners to function effectively in shade selection, they must meet two critical criteria: precise color imaging and robust data processing. More precisely, the accuracy of tooth color measurement depends primarily on two critical variables: the spectral characteristics of the illumination source and the chromatic analysis algorithms employed by the software—both of which reflect the fundamental factors governing imaging accuracy in digital cameras. Although these devices can record tooth shade and 3D topographic data, inherent factors may introduce variability, thereby limiting their color measurement accuracy.

Traditional colorimetric methodologies, which depend on either human visual assessment or tristimulus value quantification, are susceptible to variations in lighting conditions and observational environments, and are inherently incapable of circumventing the challenges posed by metamerism. Traditional RGB cameras and colorimeters capture limited color information, whereas spectral reflectance measurements offer a precise and objective method for assessing the optical properties of dental structures across various wavelengths by quantifying the ratio of reflected light to incident illumination. This technique effectively minimizes the influence of external variables such as light-source intensity, spectral composition, and environmental conditions.

**Spectral analysis**. Spectral analysis is a high-precision technique for color and material characterization, capable of capturing detailed reflectance information across multiple wavelengths. In dental color measurement, spectral analysis is grounded in the optical properties of teeth and involves measuring their spectral reflectance across the visible spectrum (typically 380–780 nm) to accurately characterize tooth color. The primary instrument employed for spectral analysis is the spectrophotometer.

A spectrophotometer is an analytical instrument that measures the spectral reflectance or transmittance curve of a specimen, or reflected by a material as a function of wavelength [16]. Spectrophotometers measure the amount of light energy reflected from an object at 1–25 nm intervals along the visible spectrum [17]. It contains a source of optical radiation, a means for dispersing light, an optical system for measuring and detecting, and a means for converting light obtained to a signal that can be analyzed [18]. In modern shade assessment, spectrophotometers are widely used as objective instruments. They typically employ either a tungsten-filament bulb or an LED lamp as the white-light source, delivering continuous illumination across the visible spectrum (400–700 nm) to ensure accurate color measurement. This polychromatic light is diffracted by a prism into discrete wavelength bands (10–20 nm bandwidth), which then interact with the sample through reflection, transmission, or scattering phenomena. Compared with observations by the human eye, or conventional techniques, it was found that spectrophotometers offered a 33% increase in accuracy and a more objective match in 93.3% of cases [8]. While spectrophotometers provide high precision and objectivity in dental color measurement, their high cost, operational complexity, sensitivity to measurement conditions, and poor portability limit their widespread use in clinical practice.

As previously demonstrated, existing methods encounter one or more of the following challenges: time-consuming procedures, metamerism, subjective measurements, high costs, and operational complexity. To address the limitations outlined above, this investigation proposes a deep-learned, end-to-end spectral reflectance prediction framework (SRNet) for reconstructing the spectral reflectance of teeth from RGB images under complex illumination conditions. A custom optical imaging system was independently developed, and a comprehensive training dataset comprising 4000 paired samples was generated, each containing synchronously captured RGB images and their corresponding hyperspectral image cubes. The spectral characteristics of both the illumination source and dental substrates were integrated through channel-wise fusion, and a physically interpretable neural network was proposed to predict the spectral reflectance of tooth structures. During training, different scene spectra features supplied by different components are jointly learned, and the mapping relationship between RGB image and hyperspectral image can be automatically built. Prior to analysis, two reference images must be acquired: (1) the target object under standardized illumination conditions, and (2) the light source reflected from a calibrated white reference board. These paired inputs enable the neural network to accurately compute the object’s spectral reflectance. Additionally, a customized loss function, referred to as the structure–pixel loss, together with attention mechanisms, is employed to enhance the performance of the network. Experiments validate the proposed approach on real captured data, and shows precise recovery of spectral reflectance.

Our paper provides the following key contributions:

(1) For accurate spectral reflectance prediction and color measurement, an end-to-end, physically interpretable multi-module deep learning framework (SRNet) is developed, which reconstructs spectral reflectance from RGB images under complex lighting conditions, enabling color measurement and diffuse spectrum matching between natural teeth and ceramics across multiple scenes, advancing spectral imaging network design and optimization.
(2) A dataset containing 4000 RGB–hyperspectral image pairs was built and tailored to the experiment setup consisting of a programmable illumination box featuring LED arrays with adjustable intensity and chromaticity and a dual-mode optical system incorporating a beam splitter to simultaneously capture hyperspectral image cubes and RGB images.

## 2. Methodology

The proposed approach aims to reconstruct spectral reflectance from its corresponding RGB representation, formulating hyperspectral image (HSI) recovery from spectrally sparse measurements as a spectral enhancement task. This problem is inherently ill-posed due to the substantial loss of spectral details during image acquisition, where the observed radiance results from the product of the object’s spectral reflectance and the illumination’s spectral power distribution. In the absence of explicit illumination data, the reconstruction must implicitly estimate both factors, which increases ambiguity. A light-source image, obtained by capturing a spectrally flat reference (e.g., a diffuse white target) under identical illumination, provides a direct measurement of the illumination spectrum. Incorporating this information constrains the inverse problem and mitigates spectral estimation errors, leveraging inter-band correlations in hyperspectral datasets to expand the three-channel input into a higher-dimensional spectral representation, as supported by prior findings on the critical role of illumination in reflectance reconstruction accuracy [19].

To address this challenging problem, a physics-informed, multi-model deep-learning framework “SRNet” is proposed. A beam splitter was utilized to simultaneously capture co-registered RGB and hyperspectral images of both dental specimens and illumination sources, resulting in a curated dataset comprising 4000 paired samples. The sample RGB image and the light-source RGB image are separately input into SRNet-P and SRNet-L, where they are reconstructed into their corresponding hyperspectral images. After concatenation along the channel dimension, the two hyperspectral images are further processed by the SRNet-L network under physical constraints to reconstruct the final spectral reflectance. Through the incorporation of an attention mechanism, the framework adaptively integrates multi-modal information to form a specialized spectral learning architecture, thereby facilitating precise reconstruction of spectral reflectance. A subset of the training samples is presented in Appendix A.

### 2.1. Spectral Mapping and Reconstruction Principles

An RGB image is generated by projecting a hyperspectral image (HSI) along the spectral dimension using spe_c_ific spectral response functions corresponding to the RGB channels. *S*(*x, y, λ*) represents the spectral reflectance at spatial coordinates (*x, y*) and wavelength *λ*, and *C_k_*(*λ*) denotes the camera response curve. When imaged in the human eye, *C_k_*(*λ*) becomes the visual response curve of the ocular eye. The relationship between the RGB image and *I*, *S*, *C_k_*(*λ*) can be formulated as:

(1) $$G_{1}\left(x,y\right)=\int_{380\mathrm{nm}}^{780\mathrm{nm}}I\left(\lambda \right)S\left(x,y,\lambda \right)C_{k}\left(\lambda \right)d\lambda$$

where *k* ∈ {*R, G, B*} is defined as the spectral channel index, and *I*(*λ*) refers to the spectral power distribution of the light source.

When the light source illuminates a standard diffuse whiteboard, its spectral reflectance is equal to 1, since the whiteboard is considered an ideal diffuse reflector. The relationship can be expressed as:

(2) $$G_{2}\left(x,y\right)=\int_{380\mathrm{nm}}^{780\mathrm{nm}}I\left(\lambda \right)C_{k}\left(\lambda \right)d\lambda \left(k\in R,G,B\right)$$

At the same time, Equations (1) and (2) are transformed into their discrete vector–matrix representation, given by:

(3) $$G_{1}\;\left(x,y\right)=\underset{\lambda }{\sum }I\left(\lambda \right)S\left(x,y,\lambda \right)C_{k}\left(\lambda \right)$$

(4) $$G_{2}\;\left(x,y\right)=\underset{\lambda }{\sum }I\left(\lambda \right)C_{k}\left(\lambda \right)$$

The training dataset is generated based on Equations (3) and (4), with further details provided in Section 3.

The acquisition of *I*(*λ*)*S*(*x*, *y*, *λ*) and *I*(*λ*) can be discretized as follows by employing SRNet:

(5) $$G_{1}\;\left(x,y\right)\overset{SRNet-P}{\to }I\left(\lambda \right)S\left(x,y,\lambda \right)$$

(6) $$G_{2}\;\left(x,y\right)\overset{SRNet-L}{\to }I\left(\lambda \right)$$

(7) $$G_{1}\left(x,y\right)⊗G_{2}\left(x,y\right)\overset{SRNet-L}{\to }S\left(x,y,\lambda \right)$$

In Equation (7), the symbol ‘⊗’ denotes concatenation of the two inputs along the channel dimension.

According to Equations (3) and (4), the projection from HSI to RGB contains two main steps: (1) spectral response encoding and (2) integration summation, in which the 3D hyperspectral cube is compressed into a 2D image. Therefore, the HSI can be completely rebuilt from each channel of the RGB image by solving a compressive-imaging inverse problem.

**Figure 1:**
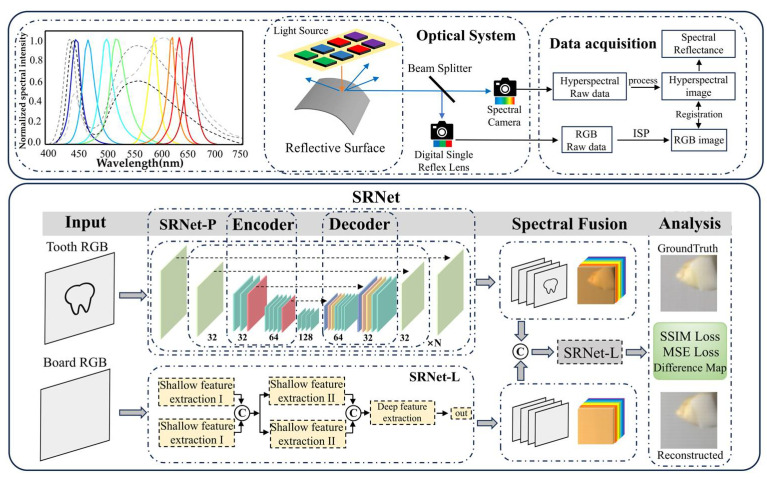
Overview of the proposed SRNet framework, which takes RGB images and light-source images as inputs and outputs spectral reflectance. The framework consists of two subnetworks, SRNet-P and SRNet-L. SRNet-P reconstructs hyperspectral data from the tooth images, while SRNet-L converts light-source images into hyperspectral data. Physical information fusion is achieved by aligning and concatenating the two hyperspectral outputs along the channel dimension. The final spectral reflectance is then obtained by further processing the fused spectral data through SRNet-L. The orange and blue arrows represent incident light from the light box and reflected light, respectively. The black and gray arrows indicate the transition to subsequent steps. The network architecture is introduced in Section 2.2, and the optical system and dataset construction are detailed in Section 3. The letter “C” denotes concatenation of the two inputs along the channel dimension. *N* indicates the number of repeated modules. The black dashed arrows between modules indicate skip connections.

The reconstruction process is formulated to reconstruct the original data from its observed projections: *G*_1_(*x*, *y*) → *I*(*λ*)*S*(*x*, *y*, *λ*), *G*_2_(*x*, *y*) → *I*(*λ*). Through *I*(*λ*)*S*(*x*, *y*, *λ*) and *I*(*λ*), we can obtain the final spectral reflectance. To manage this problem, the proposed SRNet framework integrates two dedicated modules: the SRNet-P block and the SRNet-L block. The schematic representation of the method is presented in Figure 1. The network receives a paired RGB image and light-source image as input and outputs the reconstructed spectral reflectance corresponding to the scene.

**Step 1**: *G*_1_(*x*, *y*) → *I*(*λ*)*S*(*x*, *y*, *λ*)

According to Equation (5), SRNet-P is tasked with reconstructing the hyperspectral image from the RGB image of the tooth sample. SRNet-P adopts a U-Net-like network architecture. Rather than performing upsampling via simple interpolation, the U-Net structure progressively upsamples the RGB image to the target spectral resolution and maps these features into a specific representation, enabling the generation of *I*(*λ*)*S*(*x*, *y*, *λ*) based on the unique spectral characteristics of the scene. To further mitigate the impact of noise and improve reconstruction accuracy, attention modules are incorporated into the U-Net-like structure within SRNet-P, allowing the network to extract salient spectral features and integrate them with highly correlated contextual information.

**Step 2**: *G*_2_(*x*, *y*) → *I*(*λ*)

As indicated by Equation (6), our goal remains to reconstruct hyperspectral images from RGB images. In SRNet-L, convolutional layers are used in place of fixed upsampling operations to better extract image information. Unlike fixed operations such as max pooling and interpolation, convolutional layers can adaptively learn spatial and semantic relationships through training, thereby preserving more contextual and structural information. This substitution not only enables more flexible and fine-grained feature extraction and reconstruction, but also facilitates the integration of attention mechanisms and physical priors, ultimately improving the overall performance of the network.

**Step 3**: *I*(*λ*)*S*(*x*, *y*, *λ*) ⊗ *I*(*λ*) → *S*(*x*, *y*, *λ*)

Prior to Equation (7), SRNet-P reconstructs the hyperspectral image from the tooth sample’s RGB image, while SRNet-L reconstructs the hyperspectral image from the light-source image. These two hyperspectral outputs are concatenated along the channel dimension and then fed back into SRNet-L to extract spectral information for decoding, ultimately yielding the final spectral reflectance.

The ”⊗” in inversion step 3 indicates that the hyperspectral data of the light source and the hyperspectral images of the teeth are concatenated along the channel dimension. During backpropagation, the network parameters are optimized by minimizing the loss between the reconstructed hyperspectral image (HSI) and the ground-truth HSI. The detailed internal architectures of each submodule are presented in Section 2.2.

### 2.2. SRNet

#### 2.2.1. SRNet-P

**Figure 2:**
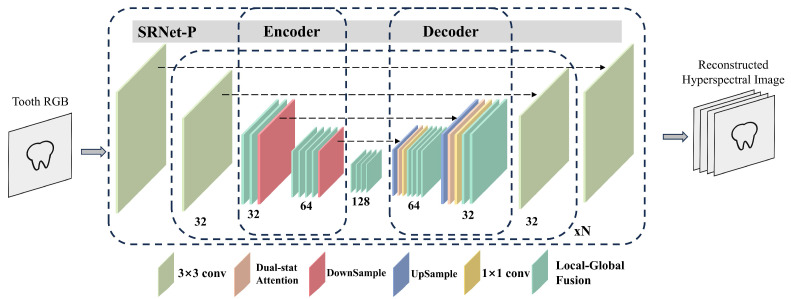
Detailed architecture of SRNet-P, mapping an RGB image input to a hyperspectral image output. The numbers below each layer denote its output dimensions. *N* indicates the number of repeated modules. The modules shown beneath the diagram are color coded: for example, blue represents upsampling layers and red represents downsampling layers. The black dashed arrows between modules indicate skip connections.

U-Net [20] is a widely adopted architecture in image segmentation and recognition tasks, characterized by its encoder–decoder structure with symmetric downsampling and upsampling paths. A distinctive feature of U-Net is its skip connections, which facilitate the transfer of fine-grained, low-level features directly to the upsampling layers, thereby mitigating model degradation and preserving spatial details. The downsampling convolutions enable extraction of high-dimensional representations, which are subsequently decoded and classified through upsampling operations. The architecture of SRNet-P, as proposed in our SRNet framework and illustrated in Figure 2, adopts a similar design. It accepts an RGB image as input and reconstructs a hyperspectral image with spatial resolution *H × W* and spectral dimension *C*.

SRNet-P takes an input feature map *X_in_* ∈ *R^B×C×H×W^* and first projects it into a latent feature space via a 3 × 3 convolution:

(8) $$F_{0}=conv_{3\times 3}(X_{in})$$

The encoded features then pass through multiple hierarchical stages, each comprising several local–global fusion (LGF) extraction modules followed by a downsampling convolution, progressively capturing spatial and spectral representations.

(9) $$F_{i}=DownConv(LGF_{K_{1}}(F_{i-1})),i=1,2,3\ldots N$$

where *K* in *LGF_k_*(·) indicates k refers to the number of consecutively applied LGF layers and *N* denotes the number of downsampling and upsampling in the stage.

LGF modules in feature extraction further refines the deepest features before the decoder reconstructs the spatial resolution through transposed convolutions:

(10) $$F_{FE}=LGF_{K_{2}}(F_{N})$$

At each decoding stage, features are fused with corresponding encoder outputs enhanced by a dual-stat attention mechanism, allowing the network to emphasize relevant spectral information.

(11) $$S_{i}=UpConv(F_{N-i})$$

(12) $$S_{i}=Conv_{1\times 1}(Concat(S_{i},DSA(F_{N-i})))$$

(13) $$F_{N-i-1}^{\prime }=LGF_{K_{3}}(S_{i})$$

where *Concat*(*A*, *B*) denotes concatenation along the channel dimension.

Finally, a convolutional layer maps the features back to the original dimension, and a residual connection adds the input to the output, yielding the reconstructed output:

(14) $$P=conv_{3\times 3}(F_{0}^{\prime })+X_{in}$$

This architecture effectively integrates multi-scale feature extraction, attention, and residual learning to improve hyperspectral reconstruction accuracy.

The LGF (local–global fusion) module and DSA (dual-stat attention) module are described in Appendix B.

#### 2.2.2. SRNet-L

**Figure 3:**
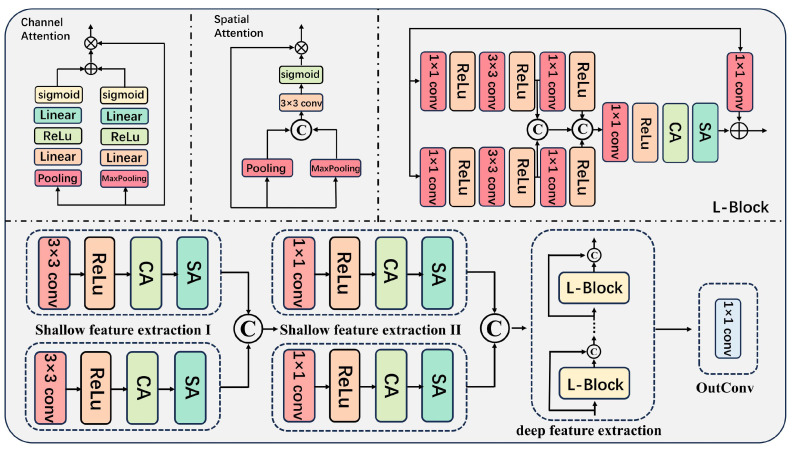
Detailed structure of SRNet-L. It takes an RGB image as input and outputs spectra with 32 channels. The network consists of three main modules: shallow feature extraction, deep feature extraction, and attention mechanism. In Figure 3, ⊕ denotes element-wise addition, ⊗ denotes multiplication, and “C” indicates concatenation along the channel dimension of the two inputs.

SRNet-L is designed to reconstruct the light-source spectra from an RGB image and to decode the fused spectral information. The structure introduced in our SRNet is demonstrated in Figure 3. It takes the RGB image as input and outputs the raw hyperspectral image with *H × W* spatial resolution and *C* spectral bands. It processes the input RGB image of the light source to recover the underlying light-source spectral information, enabling the decoding of the fusion spectra. Spatial attention (SA) and channel attention (CA) mechanisms are applied separately to model long-range semantic dependencies across spatial locations and spectral bands, respectively. The L-block employs a residual learning strategy through a convolutional shortcut connection, facilitating element-wise addition between the attention-weighted features and the linearly transformed input features. This architectural design not only preserves intrinsic spectral characteristics throughout the network depth but also mitigates gradient vanishing, thereby enhancing training stability while maintaining spectral information integrity during hyperspectral reconstruction. This architecture effectively extracts and refines shallow features through attention mechanisms and recursive L-blocks, facilitating the generation of light-source hyperspectral imagery with high spatial and spectral fidelity.

The input to the network is a feature tensor *X_in_* ∈ *R^B×^^C×^^H×^^W^,* where *B*, *C*, *H*, and *W* represent batch size, number of channels, height, and width, respectively. Initially, shallow feature extraction is performed through two parallel convolutional paths, each consisting of a 3 × 3 convolution followed by ReLU activation, channel attention (CA), and spatial attention (SA) modules, producing feature maps *F*_1_ and *F*_2_:

(15) $$F_{1}=SA(CA(\mathrm{ReLu}(Conv_{3\times 3}(X_{in}))))$$

(16) $$F_{2}=SA(CA(\mathrm{ReLu}(Conv_{3\times 3}(X_{in}))))$$

These initial features are then further refined by applying 1 × 1 convolutions, ReLU, and the same attention mechanisms, yielding *F*_3_ and *F*_4_:

(17) $$F_{3}=SA(CA(\mathrm{ReLu}(Conv_{1\times 1}(F_{1}))))$$

(18) $$F_{4}=SA(CA(\mathrm{ReLu}(Conv_{1\times 1}(F_{2}))))$$

Subsequently, these four feature maps are concatenated along the channel dimension to form a fused feature representation:

(19) $$F_{fusion}=Concat(F_{1},F_{2},F_{3},F_{4})$$

To enhance the fused features, a sequence of N L-blocks—each comprising multiple convolutional layers, attention modules, and residual connections—is recursively applied:

(20) $$F_{i+1}=L_{i}(F_{i}),\;i=0,1,2,\ldots ,N-1,\;F_{0}=F_{fusion}$$

The output after the final L-block, *F_out_ = F_N_,* is then projected via a 1 × 1 convolution to the desired output *L_out_*:

(21) $$L_{out}=Conv_{1\times 1}(F_{out})$$

### 2.3. Loss Function

To ensure the stability of the training process, a tailored loss function is introduced that accounts for the spatial locations. The disparity between the ground-truth and the reconstructed hyperspectral image (HSI) is evaluated using different loss components. The structure–pixel loss function, *L_overall_*, can be described as follows:

(22) $$L_{overall}=\alpha L_{MSE}+\beta L_{SSIM}$$

where *L_MSE_* denotes the mean squared error (MSE) used to quantify the pixel-wise difference, and is defined as follows:

(23) $$L_{MSE}=\frac{1}{M}\overset{M}{\underset{i=1}{\sum }}||H_{i}^{*}-H_{i}|{|}^{2}$$

where *M* denotes the total number of pixels in the hyperspectral image (HSI), while H* and *H* correspond to the pixel values of the reference (ground-truth) and the reconstructed HSI, respectively.

During the training process, to ensure both the integrity of the overall structure and the accuracy of individual pixels, we set *α* and *β* to 1. The MSE (mean squared error) loss function enforces precise spectral reconstruction through direct pixel-wise penalization of deviations between reconstructed and ground-truth reflectance values.

*L_SSIM_* = 1 − *SSIM*(*x*, *y*), where *SSIM*(*x*, *y*) denotes the local structural similarity index between *x* and *y*. This metric evaluates the consistency of luminance, contrast, and structural information between the reconstructed image and the ground-truth. The *SSIM*(*x*, *y*) is defined as:

(24) $$SSIM\left(x,y\right)=\frac{\left(2\mu_{x}\mu_{y}+C_{1}\right)\left(2\sigma_{xy}+C_{2}\right)}{\left(\mu_{x}^{2}+\mu_{y}^{2}+C_{1}\right)\left(\sigma_{x}^{2}+\sigma_{y}^{2}+C_{2}\right)}$$

where *C*_1_ and *C*_2_ are predefined parameters assigned values of 0.0001 and 0.0009 based on empirical evaluation. *μ_x_* represents the average intensity of *x*.

The variables *σ_x_*^2^ and *σ_xy_* are the variance of *x* and the co-variance of *x* and *y*. *μ_y_* and *σ_y_^2^* represent the average intensity and variance of *y*, respectively. Furthermore, *L_SSIM_* is bounded within the interval [0, 1], owing to the fact that *SSIM* (*x, y*) itself ranges between 0 and 1.

The SSIM loss function evaluates local image patches through three complementary metrics: (1) luminance consistency, (2) contrast preservation, and (3) structural correlation. This multi-dimensional assessment effectively maintains the spatial–spectral coherence of reconstructed spectra, preserving both inter-band correlations and intra-band spatial transitions.

When reconstructing high-dimensional spectral reflectance from RGB or low-resolution spectral inputs, the joint loss function effectively prevents spectral distortion by simultaneously optimizing for both local accuracy and global spectral fidelity. This composite loss combines complementary error metrics to maintain the physical plausibility of recovered spectra while preserving fine spectral features.

## 3. Experiment

### 3.1. Experiment System

As shown in Figure 1, each training pair comprises five components: an RGB image of the tooth and corresponding ground-truth hyperspectral image, an RGB image of the light source reflected by a standard whiteboard captured by the same camera, the corresponding ground-truth hyperspectral image, and the ground-truth hyperspectral image of spectral reflectance. The ground-truth hyperspectral image of spectral reflectance is computed from the sample and light-source hyperspectral images, retaining the same spatial dimensions as the originals and representing the intrinsic spectral properties of the sample. The detailed computation procedure is provided in Section 3.2.3.

**Figure 4:**
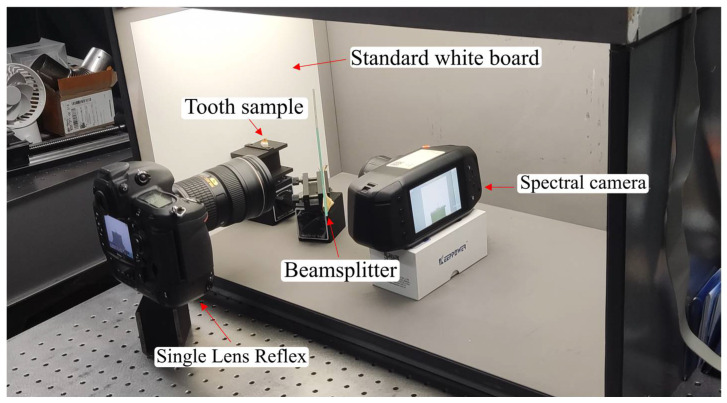
Experiment setup to capture the hyperspectral images and RGB images.

To maintain uniformity in the experimental input data pairs and enhance the robustness of the spectral reconstruction framework, the optical system was designed and assembled in accordance with the following specifications. Instead of utilizing the pre-existing simulated dataset, a binocular imaging system was designed and implemented to acquire a real-world dataset. A beam splitter was employed to divide the incident light into two separate beams, enabling the simultaneous acquisition of ground-truth and corresponding images sharing an identical field of view. As depicted in Figure 4, the light reflected from the object is incident on the semi-transparent, semi-reflective beam splitter at a 45-degree angle. This arrangement guarantees that the reflected image is aligned perpendicular to the optical axis of the DSLR camera (Nikon Corporation, Tokyo, Japan), while the transmitted image is oriented perpendicular to the optical axis of the spectral camera lens. Both devices were calibrated and securely mounted to ensure a stable and consistent field of view throughout data acquisition. To aid readers in comprehending the essential details of the equipment and materials used in the experimental setup, the key information has been consolidated into a table. For further details, please refer to Appendix C.

#### 3.1.1. Collection and Preparation of Tooth Samples

The collection and preparation of tooth specimens for this investigation were conducted at the West China Hospital of Stomatology, Sichuan University. Restorative specimens were fabricated by bonding custom-designed ceramic laminates onto extracted human teeth.

For the ceramic component, presintered white zirconia discs (comprising ZrO_2_, HfO_2_, and Y_2_O_3_ > 99%; Al_2_O_3_ < 0.5%; with a Y_2_O_3_ content of 3 mol%) were utilized. The ceramic laminates were designed employing computer-aided design and manufacturing (CAD/CAM) technology, incorporating the anticipated shrinkage ratio during the sintering process. Milling of the presintered discs was performed using a dental ceramic milling apparatus (AMW-500, Aidite, Qinhuangdao, China), followed by sintering in a rapid sintering furnace (CSF 200, Aidite, Qinhuangdao, China). To offset material loss during polishing, the initial thickness of the specimens was fabricated to be 0.05 mm greater than the target final dimension.

Surface finishing was executed via an automated polishing system (Tegramin-30, Struers, Struers A/S, Ballerup, Denmark.). Sequential polishing employed silicon carbide abrasive papers with grit sizes of 320, 600, and 1500, utilizing water as the polishing medium. Each polishing step lasted 16 s at a rotational speed of 1500 rpm under unidirectional rotation. Subsequent polishing was conducted using diamond suspensions with particle sizes of 5 μm and 2 μm (Fuyun Technology, Shanghai, China), applied with canvas and silk polishing cloths, respectively, for 20 s at 1500 rpm under identical rotational conditions. Post-polishing, residual abrasive particles were removed by rinsing with distilled water, and samples were dried using compressed air prior to storage in screw-cap tubes. The final ceramic specimen thickness ranged between 0.4 mm and 2.0 mm.

Natural tooth specimens were collected from patients at the West China Hospital of Stomatology during the period from 1 December to 31 December 2023. Teeth (including premolars and molars) were extracted due to severe periodontitis or orthodontic indications. The collection protocol was approved by the institutional ethics committee (Approval No. WCH-SIRB-2022-257). Inclusion criteria mandated the absence of visible attrition, wear, carious lesions, restorations, developmental anomalies, or pronounced discoloration. Following extraction, teeth were cleaned to remove calculus and residual periodontal soft tissue, rinsed with physiological saline, and stored in a 0.1% thymol solution.

To fabricate the final restorative specimens, zirconia ceramic laminates were bonded to the prepared natural teeth. Enamel and superficial dentin from the buccal or labial surfaces were removed using a high-speed dental handpiece (T4, Dentsply Sirona) equipped with a diamond bur operating at 1200 rpm under continuous water cooling. The resulting tooth fragments were cleansed with 75% ethanol, air-dried using compressed air, and stored in screw-cap containers. The prepared tooth surfaces were then desiccated, and dual-cure resin cement (U200, 3M ESPE, Seefeld, Germany) was applied. Each zirconia laminate was carefully positioned onto the conditioned surface to ensure intimate contact without air entrapment. Excess cement was gently removed with a cotton swab, followed by light curing with a 565 nm LED curing unit (Philips, Shenzhen, China) for 20 s to complete the bonding process and finalize the restorative specimens.

#### 3.1.2. Light Box

To ensure the robustness of the model and reflect practical usage scenarios, an independent experimental light source in the form of an LED lighting box was designed, as illustrated in Figure 4. This lighting box primarily comprises three components: the light-emitting unit, the diffusion mechanism, and the control system. The light-emitting unit contains sixteen LEDs labeled 1 through 16. Among these, LEDs numbered 6, 11, and 16 emit warm white light with varying color rendering indices; LEDs 7, 10, and 13 produce neutral white light with different color rendering capabilities; and LEDs 1 and 4 generate cool white light with distinct color rendering characteristics. The remaining LEDs emit monochromatic light across various hues, resulting in a total of eleven distinct LED types. Beneath the LED array, a diffusion panel was placed to achieve homogeneous lighting through the scattering of photons spanning multiple wavelengths. The control system consists of a programmable device connected to the circuit board via a data interface, allowing reprogramming of the LED control software to modulate the output spectral characteristics of the LEDs. Details of the light box can be found in Appendix D.

#### 3.1.3. Camera Specifications

Hyperspectral images were acquired using a Specim-IQ handheld imager (Spectral Imaging Ltd, Oulu, Finland), which captures spectral data across 204 bands within the wavelength range of 397.32–1003.58 nm, together with corresponding reference white calibration data. The resulting hyperspectral dataset includes source files with a spatial–spectral resolution of 512 × 512 × 20 and reference white files of size 512 × 1 × 204, which were subsequently employed for further data processing. For RGB image acquisition, a Nikon D3X digital single-lens reflex (DSLR) camera(Nikon Corporation, Tokyo, Japan) was employed. To minimize potential interference from subsequent algorithmic processing, the camera was configured to capture unmosaicked RAW images, producing single RGB images at a resolution of 6048 × 4032 pixels.

#### 3.1.4. Geometrical Light Path

**Figure 5:**
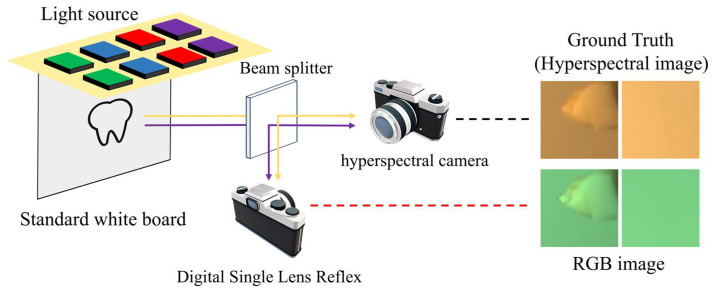
Schematic representation of the experimental imaging setup. The scene light is divided into two paths after a beam splitter. The yellow lines indicate the light-source pathways, while the purple lines represent the optical paths associated with the teeth. After passing through the beam splitter, the light is directed into both the hyperspectral and single-lens reflex cameras for imaging, thereby simultaneously capturing the RGB and hyperspectral images required for the dataset. The red and black dotted lines in the figure indicate the images acquired by the respective cameras.

In the experimental setup, the optical layout was designed to enable the simultaneous acquisition of spectral and RGB data from the same object under identical illumination. As shown in Figure 5, the light reflected from the sample surface first encounters a beam splitter inclined at 45°, which exhibits both reflective and transmissive properties. This component divides the light into two separate optical paths: one path is reflected at a right angle toward the digital single reflex lens camera, while the other passes straight through to the hyperspectral imaging unit. With this arrangement, both cameras observe exactly the same area of the target within an identical field of view, which effectively reduces spatial mismatch and ensures consistent data across both imaging modalities. Such a configuration is essential for reliable multi-source data integration, spectral characterization, and precise visual reconstruction.

### 3.2. Dataset Acquisition

The experimental system generated a theoretically maximum of 1600 unique image datasets (16 light sources × 20 tooth surfaces × 5 replicates), where each tooth’s buccal and lingual surfaces were restored with ceramic veneers of three distinct thickness gradients.

#### 3.2.1. Light Condition

For dataset construction, and considering the wide spectral variations in typical lighting conditions, sixteen distinct LED combinations were employed to simulate real-world light sources. These combinations are classified into four primary categories: (1) monochromatic lighting, which includes three variants of warm white, three of neutral white, and two of cool white LEDs; (2) binary white–white mixed lighting, encompassing mixtures such as neutral white LED 7 combined with warm white LED 6, warm white LED 6 mixed with neutral white LED 1, neutral white LED 7 combined with cool white LED 1, neutral white LED 7 mixed with cool white LED 4, and warm white LED 6 combined with cool white LED 4; (3) tricolor white mixtures, represented by two combinations—neutral, warm, and cool white mixes 7-6-1 and 7-6-4; and (4) multicolor mixed lighting, which involves an eleven-LED combination (LEDs 1, 2, 3, 5, 6, 7, 8, 9, 12, 14, and 15) generating a complex mixed white light. Details of the light box can be found in Appendix D.

#### 3.2.2. Image Preprocessing and Augmentation

**Figure 6:**
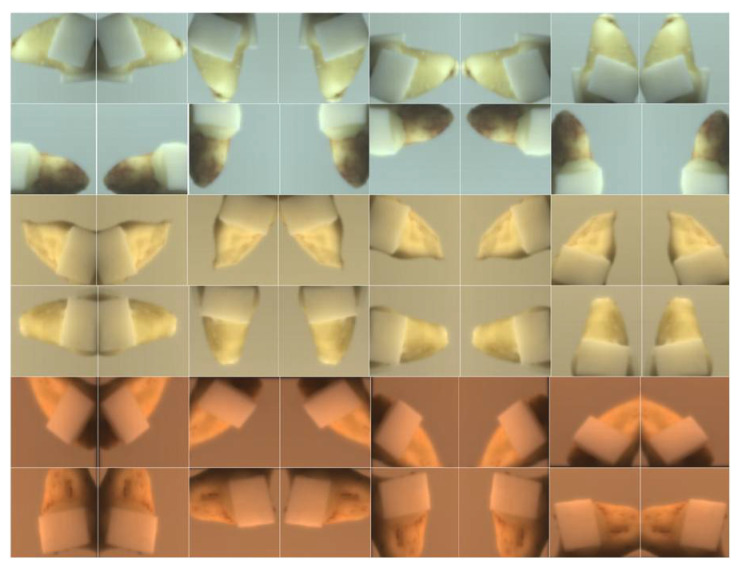
Representative dental samples under different illumination spectra, with varying ceramic thicknesses and augmented viewing angles.

A series of ISP procedures, including black-level correction and bilinear interpolation demosaicking, were applied to produce linear RGB images. Because the DSLR camera and hyperspectral camera captured data at different resolutions, the RGB images were resampled using nearest-neighbor interpolation to align with the hyperspectral data resolution. RGB and hyperspectral images were co-registered using a checkerboard calibration method to ensure precise spatial correspondence between the two modalities. To augment the dataset, techniques such as random rotation, horizontal and vertical shifts, and cropping were performed, with all padding filled using a constant value of zero. After completing registration and augmentation, the dataset comprised 4000 pairs of images—RGB images sized 64 × 64 × 3 and hyperspectral images sized 64 × 64 × 32. Figure 6 presents examples of data processed via image enhancement.

#### 3.2.3. Spectral Reflectance Generation

In our study, the ground-truth hyperspectral image of spectral reflectance was obtained by calculating the ratio between the hyperspectral image of the sample and that of the illumination source on a wavelength-by-wavelength basis. Specifically, for each wavelength *λ*, the spectral reflectance *S*(*λ*) was computed as:

(25) $$S(\lambda )=\frac{I_{sample}(\lambda )}{I_{illumination}(\lambda )}$$

where *I_sample_*(*λ*) the measured hyperspectral intensity of the sample and *I_illumination_*(*λ*) is the measured hyperspectral intensity of the illumination (obtained from a standard white board).

Please note that during the calculation of spectral reflectance, preprocessing steps including black-level correction and spatial registration were already performed to ensure the accuracy of the obtained spectral reflectance.

### 3.3. Training Details

The SRNet model was trained using 3300 image sets—comprising 3000 for training and 300 for validation—and evaluated on an independent test set of 100 image pairs, each containing RGB images alongside their corresponding hyperspectral cubes. The model optimization was performed within the PyTorch framework (version 2.7.1, CUDA 12.8) using the Adam optimizer, with hyperparameters set to *β*_1_ = 0.9, *β*_2_ = 0.999, and a weight decay of zero. The initial learning rate was established at 4 × 10^−4^, with a batch size of 8. Training proceeded for a total of 54 epochs. Convergence was achieved at the end of this process. 

## 4. Experimental Results

In the experiment, the mean squared error (MSE) and structural similarity index measure (SSIM) calculated between the reconstructed outputs and the ground-truth were chosen as objective metrics to assess network performance. Reconstruction accuracy was analyzed both spatially and spectrally. In addition to the aforementioned method, four alternative strategies employing different loss functions and network architectures were implemented for comparative evaluation.

**Evaluation 1.** The structure–pixel loss “*L*_overall_” in Equation (8) was substituted with *L_MSE_* to examine the impact of alternative loss functions. The “*L_MSE_*” loss is expressed as:

(26) $$L_{MSE}=\frac{1}{M}\overset{M}{\underset{i=1}{\sum }}||H_{i}^{*}-H_{i}|{|}^{2}$$

where *M* denotes the total number of pixels in the hyperspectral image (HSI), while H^*^ and *H* correspond to the pixel values of the reference (ground-truth) and the reconstructed HSI, respectively.

**Evaluation 2.** To assess the contributions of the attention mechanism and the physical information fusion architecture, the attention module was completely removed from the network for ablation analysis.

**Evaluation 3.** For comparisons to state-of-the-art models, HSCNN+, AWAN, HDNet, and MST++ were employed to benchmark the performance of the proposed method.

**Evaluation 4.** Ablation experiments were conducted to evaluate the impact of removing key components and inputs from the proposed network. We removed key components—DSA, LGF, SRNet-P, SRNet-L—and illumination images to assess their impact. The pretrained model was also fine-tuned on a non-dental dataset to evaluate generalization. All experiments used consistent training and preprocessing. These studies quantify each component’s effect on spectral reconstruction.

To ensure a fair comparison, the pixel values of all images were normalized to the range [0, 1] during data preprocessing. Let *θ* represent the input image, the normalized image is calculated as:

(27) $$\theta_{norm}=\frac{\theta }{\max(\theta )}$$

where *max*(*θ*) denotes the maximum pixel value within *θ*.

### 4.1. Comparison of SRNet and CNN Baseline

The experimental results for Evaluation 1 and 2 are presented in Table 1, where the final test metrics (MSE and SSIM) for each approach were computed. The Baseline 1 convolutional neural network employed in this work is based on the original U-Net architecture, which has been extensively validated in various image reconstruction and segmentation tasks [20]. Baseline 2 corresponds to our original network with all attention mechanisms removed. This configuration retains the same overall architecture and number of layers as SRNet, but excludes the DSA module in SRNet-P and the attention modules in SRNet-L, allowing us to isolate and evaluate the contributions of attention mechanisms to reconstruction quality and structural fidelity.

Table 1 presents the number of parameters, inference time, and reconstruction performance for the proposed SRNet variants and baseline networks. All SRNet variants incorporate attention mechanisms, including DSA in SRNet-P and attention modules in SRNet-L. To assess their impact, we also evaluated Baseline 2, which retains the same architecture but with all attention modules removed. Under the same loss function, SRNet achieves a substantial improvement over Baseline 2 in SSIM (0.8129 vs. 0.643) and a lower MSE, while the increase in parameter numbers and inference time remains modest. This indicates that the computational overhead of attention mechanisms is limited, whereas their contribution to structural and perceptual fidelity is substantial. Furthermore, the trade-off between model complexity, inference speed, and reconstruction quality is evident: SRNet variants achieve superior SSIM and lower MSE at the cost of slightly increased inference time, demonstrating that the network design effectively balances reconstruction accuracy and computational efficiency for practical deployment. Detailed information regarding SRNet’s number of parameters, memory consumption, and inference time is provided in Appendix E.

**Figure 7:**
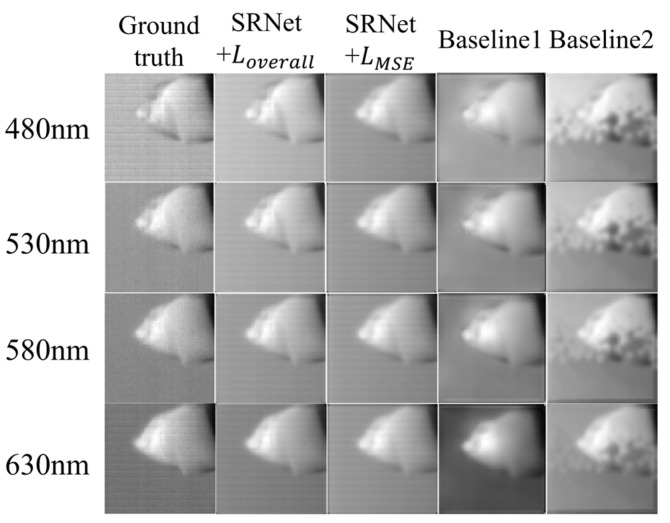
Visual comparison of Baseline 1, Baseline 2, and SRNet. Reconstruction results for four selected spectral bands (480 nm, 530 nm, 580 nm, and 630 nm) are shown.

As shown in Figure 7, schemes operating on SRNet outperform the conventional U-Net [20] structure. Due to the absence of the attention module, the results of Baseline 1 have a consistently smaller SSIM than our SRNet, which demonstrates the superiority of network architecture. However, in Baseline 2, where all attention mechanisms are removed from SRNet, the reconstructed results exhibit noticeable artifacts. The main body of the tooth is often occluded, while some fine details, such as the tooth apex, are still partially preserved. This behavior can be attributed to the substantial reduction in SRNet-L’s ability to extract and utilize light-source information without the attention modules. As a result, the information fusion process is impaired, leading not only to a failure in providing effective guidance but in some cases even hindering accurate reconstruction. This observation highlights the critical role of attention mechanisms in SRNet for capturing spatial and spectral dependencies and ensuring high-fidelity reconstruction of dental structures.

Due to the substantial performance gap in MSE and SSIM between Baseline 2 and the other methods, Baseline 2 was excluded from the comparative analysis of MSE and SSIM metrics.

**Figure 8:**
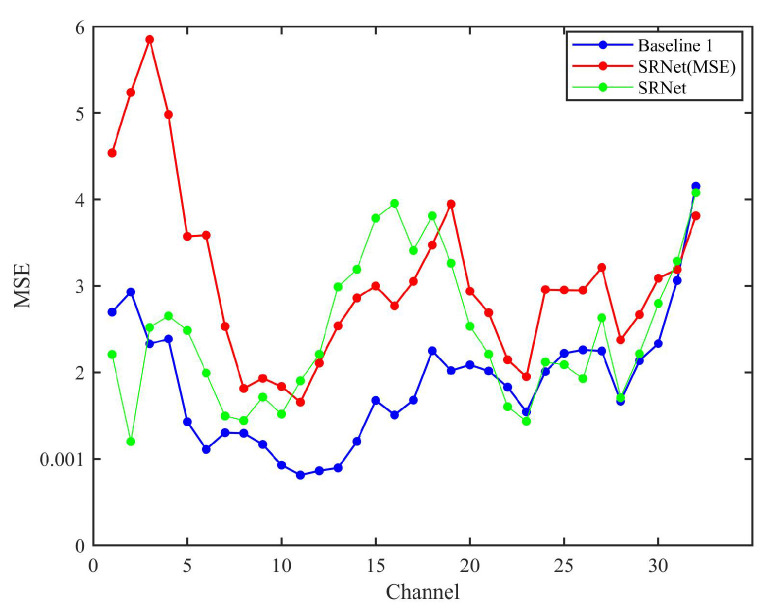
Mean squared error (MSE) performance of the three methods evaluated across the full set of 32 spectral bands. SRNet (MSE) and SRNet represent the schemes of “SRNet + *L_MSE_*” and “SRNet + *L_overall_*”, respectively.

The MSE results are illustrated in Figure 8. The reconstruction results of “*L_overall_*” exhibit superior accuracy and stability compared to those of “*L_MSE_*,” while the baseline model achieves the lowest errors in both total and mean MSE. The elevated MSE observed in SRNet relative to the Baseline 1 can be attributed to its more intricate network architecture, which entails multiple nonlinear transformations and feature fusion. Although these design choices enhance the model’s capacity to capture high-frequency details and global contextual information, they may inadvertently introduce subtle pixel-level reconstruction discrepancies that aggregate, thereby increasing the overall MSE. Conversely, the baseline model’s comparatively simpler and more direct architecture prioritizes the minimization of pixel-level errors, resulting in superior performance in terms of MSE. Under the application of an identical loss function, SRNet-MSE demonstrates a significant improvement in SSIM relative to the baseline model, indicating enhanced preservation of structural and perceptual quality. Nevertheless, its MSE remains higher than that of the baseline, which suggests that the proposed physical fusion mechanism in SRNet prioritizes the integration of global contextual information over precise pixel-level reconstruction. This trade-off highlights the model’s design focus on capturing broader spectral and spatial dependencies, which benefits overall visual fidelity at the expense of localized numerical accuracy measured by MSE. However, SRNet (MSE) fails to preserve subtle spectral structures, leading to higher errors in channels with rapid transitions. In comparison, while the standard convolutional architecture of the CNN demonstrates a certain capability in retaining such fine structures to some extent, its overall perceptual quality remains compromised due to the lack of specialized spectral modeling and limited capacity for long-range dependency modeling.

**Figure 9:**
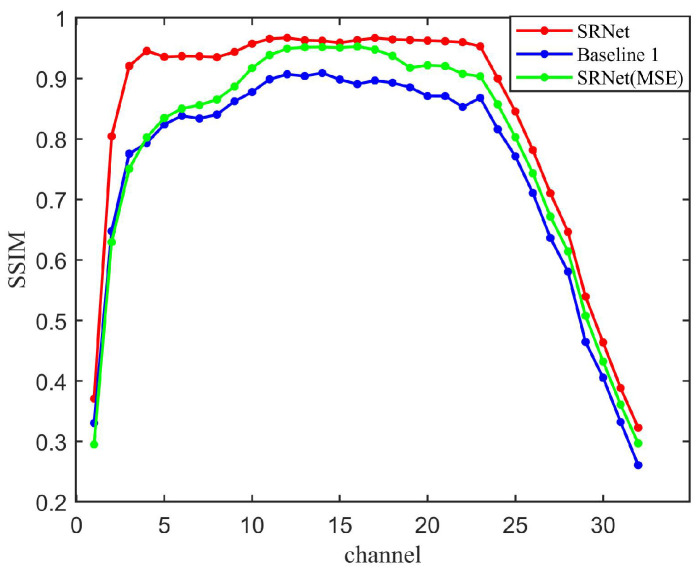
SSIM results of the three methods evaluated over all 32 spectral bands. SRNet (MSE) and SRNet represent the schemes of “SRNet + *L_MSE_*” and “SRNet + *L_overall_*”, respectively.

The SSIM results are illustrated in Figure 9. SRNet outperforms both Baseline 1 and SRNet (MSE) by 14.3% and 7.3% in mean SSIM, respectively. Furthermore, SRNet (MSE) surpasses Baseline 1, indicating that even when optimized solely for MSE, the SRNet architecture maintains superior structural fidelity compared with a standard CNN architecture. Our SRNet combines SSIM and MSE, preserving both local and global structures. However, SRNet (MSE) over-penalizes large errors but ignores perceptual quality, while Baseline 1 lacks mechanisms to prioritize physically meaningful features. It demonstrates the superiority of our loss function and physical information fusion mechanism.

### 4.2. Comparison to State-of-the-Art Methods

Our method is compared with state-of-the-art models like HSCNN+ [21], AWAN [22], HDNet [23], and MST++ [24]. Their final test results of MSE loss and SSIM are shown in Table 2. All models—including our proposed method and the comparison networks—were trained on the same dataset using an identical training protocol. The comparison models were fine-tuned on our specialized dataset to ensure adequate adaptation prior to evaluation. To guarantee fairness in the testing phase, we adjusted the input dimensions of all compared models to conform to the format and size requirements of our dataset. This adjustment was critical to ensure that all models were assessed under consistent baseline conditions, thereby enabling a fair and rigorous comparison of their performance.

**Figure 10:**
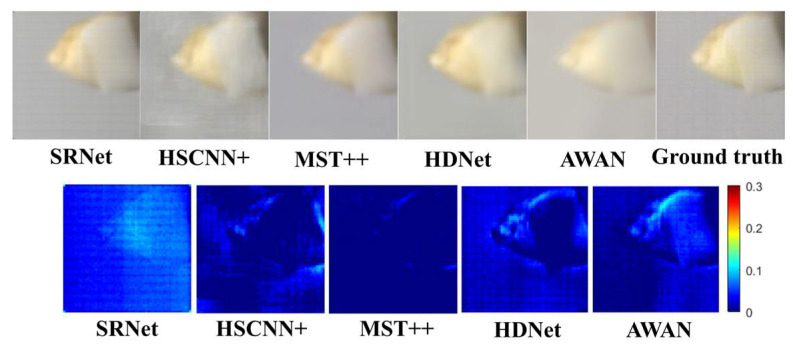
Comparative maps and difference maps of the contrast experiment. The color difference map was obtained by averaging the absolute differences between the reconstructed values and the ground-truth values across 32 wavelengths for each pixel. The comparative maps are shown above, and the difference maps are shown below.

The SSIM results demonstrate that our SRNet achieves significantly higher precision in structural similarity measurement compared to HSCNN+. Specifically, SRNet’s average SSIM score of 0.8724 substantially outperforms HSCNN+’s 0.8085, representing a 7.9% improvement in structural preservation accuracy. This precision advantage is consistent across all spectral channels, with particularly notable gains in the mid-range spectrum. In addition, the reconstructed images from different spectral channels and the corresponding difference-maps are also given in Figure 10.

In terms of reconstruction accuracy (MSE), MST++ achieves the lowest error, indicating superior pixel-level precision, likely due to its transformer-based architecture that effectively captures long-range spectral dependencies. HSCNN+ follows closely, suggesting that its CNN designs balance spectral and spatial feature extraction well. AWAN exhibits slightly higher error, possibly due to its attention mechanism focusing more on salient features rather than fine-grained reconstruction. Notably, SRNet has the highest MSE, which stems from its physics-informed structure prioritizing structural integrity over pixel-wise precision.

Conversely, in perceptual quality (SSIM), SRNet outperforms all competitors, demonstrating its strength in preserving structural details and natural image characteristics. This suggests that while SRNet may sacrifice some reconstruction fidelity (higher MSE), its physics-informed learning framework excels in maintaining high-level visual coherence. HDNet and AWAN follow, benefiting from their feature fusion and attention mechanisms, respectively. MST++ maintains competitive SSIM with its strong MSE performance, indicating a balanced approach. HSCNN+ lags behind, likely due to its simpler CNN-based architecture struggling with complex structural preservation.

As observed from the comparative maps in Figure 10, the reconstructed images using HSCNN+ exhibit noticeable background nonuniformity. In terms of edge preservation, the SRNet reconstruction demonstrates the sharpest anatomical boundaries, particularly in maintaining morphological features at the enamel–dentin junction and root apex regions, with notably superior edge definition compared to other algorithms. As evidenced by the difference maps, the MST++ reconstruction demonstrates superior pixel-wise fidelity to the ground-truth values compared to other methods. However, its performance in preserving fine edge details of dental structures and ceramic components remains slightly inferior to SRNet, as indicated by more pronounced discrepancies in these critical regions. This suggests that while MST++ achieves excellent global reconstruction accuracy, SRNet’s architecture may be better optimized for maintaining local structural details in significant areas. Regarding color reproduction fidelity, SRNet demonstrates superior performance in maintaining natural color transitions throughout the reconstructed images. In contrast, AWAN exhibits noticeable color distortion, particularly in background regions. The visual analysis further reveals MST++ suffers from over-smoothing that compromises cusp morphological details.

For overall reconstruction quality, SRNet generates images with optimal structural coherence, free from noticeable artifacts or distortions. In contrast, other algorithms display varying degrees of blurring or deformation in critical regions such as occlusal surface textures and root morphology. These qualitative observations align with and substantiate quantitative findings, collectively validating the superiority of the SRNet algorithm.

This comprehensive evaluation confirms SRNet’s advancements in three key aspects: (1) precise preservation of fine dental structures, (2) physiologically accurate color reproduction, and (3) robust avoidance of common reconstruction artifacts, establishing it as a state-of-the-art solution for dental spectral image reconstruction.

**Figure 11:**
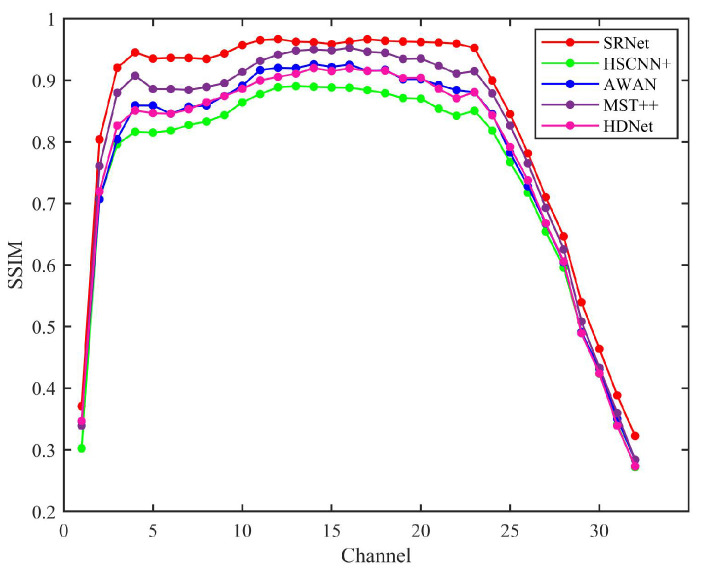
SSIM results for HSCNN+, AWAN, MST++, HDNet, and SRNet computed over all 32 spectral bands, illustrating the comparative reconstruction quality across the full spectral range.

Figure 11 illustrates the structural similarity index measure (SSIM) results obtained using various state-of-the-art methods. The SSIM evaluation demonstrates that SRNet, as a physics-informed fusion network, achieves superior structural preservation by effectively integrating physical spectral constraints with data-driven learning. Analysis demonstrates SRNet’s superior performance across all spectral channels, particularly excelling in mid-range bands where its physics-informed architecture effectively combines spectral and spatial attention mechanisms. Unlike conventional attention-based networks, SRNet maintains more consistent structural preservation throughout the spectrum. This stable performance profile suggests SRNet’s physical constraints help optimize attention weighting for more reliable feature extraction compared to purely data-driven attention approaches.

Considering SSIM metrics alone, SRNet demonstrates advantages compared to both spectral transformer-based MST++ and attention-enhanced networks such as HDNet. However, this does not imply overall superiority across all evaluation criteria. The results suggest that incorporating physical priors can help preserve the structural integrity of hyperspectral data, and that combining physical information fusion with attention mechanisms may further improve performance by guiding the network to focus on more meaningful spectral–spatial relationships.

**Figure 12:**
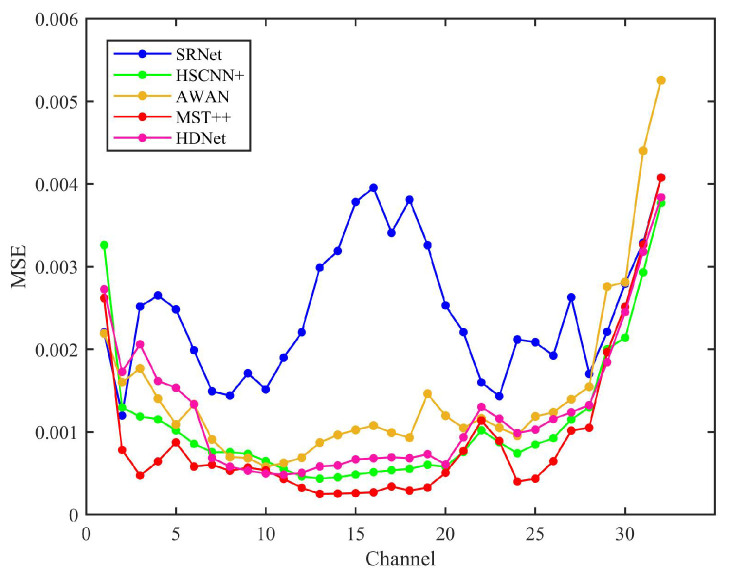
Mean squared error (MSE) results of HSCNN+, AWAN, MST++, HDNet, and SRNet evaluated across all 32 spectral bands, reflecting the overall reconstruction accuracy of each method.

Figure 12 compares the mean squared error (MSE) performance across various state-of-the-art methods. The mean squared error (MSE) analysis of state-of-the-art methods across 32 channels reveals distinct performance trends. MST++ consistently demonstrates the lowest MSE values in most channels, particularly in channels 3–20, where its MSE remains below 0.001, indicating superior accuracy. HSCNN+ and AWAN perform moderately, with HSCNN+ achieving lower MSE than AWAN in early channels but being surpassed by AWAN in certain channels (e.g., channels 8–10). HDNet and SRNet exhibit higher MSE values, with SRNet showing significant fluctuations, especially in channels 13–16, where its MSE peaks above 0.003. SRNet’s MSE spikes in channels 13–16 may stem from physical constraints of information fusion, yet its SSIM superiority implies these constraints effectively maintain inter-band relationships critical for human or downstream task perception.

In contrast, MST++’s lower MSE but weaker SSIM performance highlights a limitation of purely data-driven methods: while optimized for pixel accuracy, they may fail to capture physically plausible structures. SRNet’s hybrid approach thus offers a task-adaptive advantage—particularly in applications where perceptual quality outweighs marginal MSE gains.

### 4.3. Ablation Experiment

**Figure 13:**
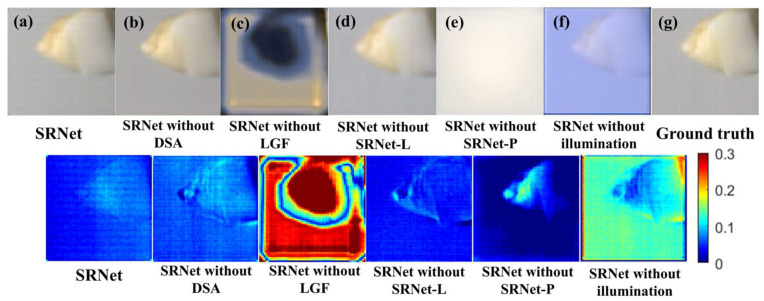
Comparative reconstruction results of various model configurations alongside the ground-truth and corresponding difference maps. The methods include (**a**) SRNet, (**b**) SRNet without the dual stat attention (DSA) module, (**c**) SRNet without the local–global fusion (LGF) module, (**d**) SRNet without the SRNet-P branch (sample input), (**e**) SRNet without the SRNet-L branch (illumination input), (**f**) SRNet without illumination input (illumination replaced by constant 1), and (**g**) ground-truth spectral reflectance. Each row presents the reconstructed spectral reflectance images and their corresponding difference maps relative to the ground-truth.

To rigorously examine the contributions and significance of individual components within the proposed network architecture, we conducted a comprehensive series of ablation experiments. In these experiments, specific modules or input data types were systematically removed to isolate their effects, and the resulting changes in reconstruction accuracy were evaluated. This approach provides deeper insights into the extent to which each element influences the overall performance of spectral reflectance prediction. Figure 13 presents the pseudo-color visualization results obtained from the ablation study.

The reconstruction results without the dual stat attention (DSA) module exhibit reduced background uniformity and diminished preservation of fine dental textures, particularly at critical regions such as the tooth roots and interfaces between teeth and ceramic components, relative to SRNet with DSA. The absence of DSA results in blurred and less distinct edges, impairing the capture of subtle morphological features.

Removal of the LGF module leads to a pronounced decline in reconstruction quality. Analysis of the pseudo-color maps and corresponding difference images reveals substantial errors throughout the scene, encompassing both dental regions and background areas, with marked deviations from the ground-truth. This deterioration arises from the pivotal role of the LGF module in SRNet-P, which is responsible for extracting essential spatial and spectral features; its absence results in the loss of critical information, including both anatomical details and background color cues. These findings highlight the indispensable function of the LGF module in maintaining accurate and detailed spectral reconstruction.

In the model variant without the SRNet-L module, dental sample images were directly used as input to generate the spectral reflectance of the teeth. The results show that using only SRNet-P achieves satisfactory overall tooth morphology, but fine details, such as tooth root shape, are less accurately reconstructed. Additionally, the reconstructed images exhibit noticeable color deviations, likely due to the absence of illumination spectral information, which leads to missing critical details in the color generation process.

Correspondingly, when employing only the SRNet-L network with illumination (light-source) images as input to reconstruct the spectral reflectance of teeth, the resulting reconstructions entirely lack dental structural details. This observation indicates that illumination information functions solely as an auxiliary input in the spectral reconstruction process. Despite the network’s capacity, it cannot infer or generate meaningful dental features from illumination data alone without prior learning from sample images.

To further examine the significance of illumination images within the proposed framework, the original illumination input was replaced with a constant value to effectively eliminate illumination information. The resulting reconstructions exhibit substantial deviations from the ground-truth. Although partial dental details are retained, the overall color distribution is markedly distorted. These findings suggest that while the SRNet-P network maintains the ability to extract dental structural features, the illumination information is indispensable as auxiliary input for accurate spectral reflectance reconstruction, and its absence critically undermines reconstruction performance.

Since the reconstruction results without the LGF module and those using only illumination images exhibit significant errors, these two methods have been excluded from the quantitative performance evaluation presented here. Results of the ablation study are presented in Table 3.

From the SSIM perspective, the complete SRNet model demonstrates the highest reconstruction fidelity, reflecting superior preservation of structural details. The model without the DSA module exhibits degraded performance compared to the full network but still outperforms the variant lacking illumination input. These results highlight the effectiveness of the proposed sample-illumination fusion strategy in enhancing structural consistency.

Regarding MSE, the SRNet-P-only model achieves values close to the full SRNet, while the variant without illumination input shows significantly higher errors. This suggests that integrating illumination information introduces optical priors that constrain the reconstruction process, potentially limiting absolute pixel-wise accuracy. Therefore, although spectral fusion enhances structural similarity, it may impose restrictions on minimizing pixel-level reconstruction error.

### 4.4. Evaluation on a ColorChecker Dataset

**Figure 14:**
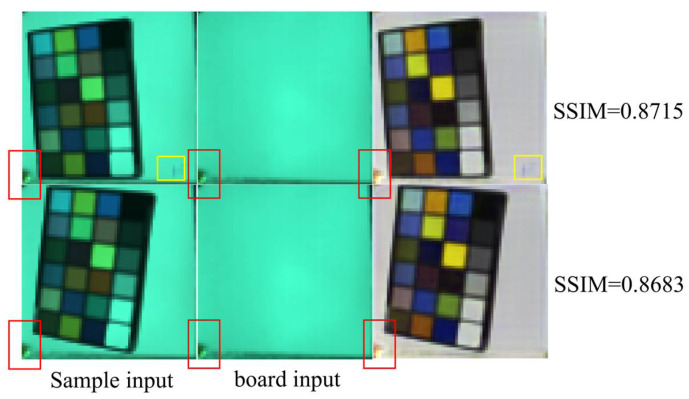
Two random test data pairs including sample input and board input. The red rectangles denote regions of nonuniform illumination, whereas the yellow rectangles indicate areas affected by random occlusions within the scene.

To investigate the generalization capability of our model on non-dental datasets, it is essential to evaluate its performance on alternative data sources. However, due to the distinctive network architecture that simultaneously requires sample RGB images and corresponding light-source RGB images as inputs, suitable publicly available datasets are limited. Given that the ColorChecker encompasses a broad spectrum of colors, evaluation using this dataset provides a meaningful indication of the model’s capability to generalize across various colors and materials. Consequently, we acquired a dedicated ColorChecker dataset to facilitate comprehensive performance assessment under these conditions. The data acquisition protocol for this dataset closely follows that of the dental dataset used in this study, with the principal distinction being that it was collected outdoors to better simulate real-world conditions. Two random test data pairs were selected, and their sample RGB-board RGB input pairs and reconstructed spectral reflectance are shown in Figure 14.

The RGB images of spectral reflectance are presented as pseudo-color renderings, while the SSIM values are calculated based on the complete reconstructed spectra. The red rectangles in Figure 14 highlight regions of nonuniform illumination, which are effectively suppressed in the output. The yellow rectangles denote occlusions present in the scene; notably, the model still reconstructs these occlusions, as they constitute genuine components of the actual spectral data. Although uncontrolled illumination and elevated noise levels contribute to a performance decline relative to the light box condition, the results remain within an acceptable range.

## 5. Discussion

Accurate color measurement is crucial for ensuring reliable sensing performance in vision-based applications. However, existing color measurement methods suffer from illumination variability, operational complexity, and perceptual subjectivity. In this study, dental color measurement, with its strict perceptual and spectral fidelity demands, is adopted to validate the proposed method. Traditional dental color measurement methods are limited by subjective perception, operational complexity, and metamerism—a phenomenon where the same color appears different under varying lighting conditions. Using self-made resin-permeated ceramic teeth, this investigation proposes a deep-learned, end-to-end spectral reflectance prediction framework to achieve snapshot teeth spectral reflectance from RGB images under complex light sources in the fundamental spectral domain through the construction of a physically interpretable network that enables physically informed feature fusion. The proposed framework consists of three key components: (1) a physically interpretable neural network for channel-wise fusion of teeth and light-source data; (2) a custom optical system within a self-designed light box; and (3) resin-infiltrated ceramics and natural teeth as samples.

Beam-splitting mirrors were utilized to simultaneously capture co-registered RGB and hyperspectral images of both dental specimens and illumination sources, resulting in a curated dataset comprising 4000 paired samples. The object’s reflected light strikes a 45° semi-transparent beam splitter, directing the reflected image orthogonally to the center of the single-lens reflex camera lens and the transmitted image orthogonally to the spectral camera lens center. Dental and light-source images are jointly fed into SRNet. The model is trained under supervision using spectral ground-truth, with channel-wise fusion enabling feature integration. The network outputs tooth spectral reflectance, achieving precise color measurement. To enhance precision, in addition to a customized loss function, the attention submodules were designed to capture correlations across different spatial locations and spectral channels. The results demonstrate that the proposed method surpasses traditional CNN architectures, with the “SRNet + *L_overall_*” approach achieving the best performance. A major advantage of the proposed method over previous techniques is that it eliminates the need for bulky equipment and stringent experimental conditions, thereby avoiding cumbersome procedures and heterochromatic artifacts, while enhancing both the efficiency and accuracy of color measurement. Comprehensive ablation and comparative experiments indicate that the proposed method attains the highest SSIM value of 0.8724, demonstrating its superior performance in maintaining structural fidelity. Nevertheless, the MSE of 0.0024 remains higher than that of MST++, indicating that the method is not yet optimal in terms of absolute pixel-level accuracy. This shortcoming may limit its applicability in certain clinical scenarios where stringent pixel-level precision is required. Addressing this limitation will constitute an important focus of subsequent research.

From a technical perspective, this work offers a straightforward, end-to-end, and scan-free solution for accurate color measurement. Our method effectively balances reconstruction quality and speed, making it versatile enough for diverse imaging scenarios. For high-precision color measurement, integrating camera response characteristics into the network architecture may improve spectral reconstruction accuracy. However, this approach may increase model parameters, prolong training duration, and reduce inference speed. We believe that this approach will inspire future research in multispectral imaging and support a broad range of applications.

## 6. Conclusions

In this study, we developed an a deep-learned, end-to-end spectral reflectance prediction framework to achieve snapshot teeth spectral reflectance from RGB images under complex light sources in the fundamental spectral domain through the construction of a physically interpretable network that enables physically informed feature fusion. A dual-attention modular-information fusion neural network is developed to recover the spectral reflectance directly from the RGB image for natural teeth and ceramics across multiple scenarios. A dataset containing 4000 RGB–hyperspectral image pairs was built using a self-designed optical system with complex illumination conditions.

Our network achieved an SSIM of 0.8724, a substantial improvement over the 0.8085 achieved by the HSCNN+ model. For overall reconstruction quality, SRNet generates images with optimal structural coherence, free from noticeable artifacts or distortions. In contrast, other algorithms display varying degrees of blurring or deformation in critical regions such as occlusal surface textures and root morphology. Our method effectively balances reconstruction quality and speed, making it versatile enough for diverse imaging scenarios. For high-precision color measurement, integrating camera response characteristics into the network architecture may improve spectral reconstruction accuracy. However, this approach may increase model parameters, prolong training duration, and reduce inference speed.

## Figures and Tables

**Table 1:** Quantitative comparison, parameter count, and inference time between the Baseline 1 and SRNet variants. Lower MSE denotes superior accuracy, whereas higher SSIM reflects improved structural fidelity.

Method	Loss Function	Params (M)	Inference Time (ms)	MSE ↓	SSIM ↑
Baseline 1	MSE	31.04	**3.008**	**0.0018**	0.7631
Baseline 2	MSE	**12.816**	36.8	0.012	0.643
SRNet (*L_MSE_*)	MSE	12.832	54.22	0.0031	0.8129
SRNet (*L_overall_*)	Structure–Pixel Loss	12.832	55.12	0.0024	**0.8724**

Note: ↑ indicates higher values are better; ↓ indicates lower values are better. Bold values indicate the best performance.

**Table 2:** MSE and SSIM of state-of-the-art methods and SRNet.

	Method	MST++	HSCNN+	AWAN	HDNet	SRNet (Ours)
Metrics						
MSE		**0.0009**	0.0011	0.0014	0.0012	0.0024
SSIM		0.8409	0.8085	0.8152	0.8245	**0.8724**

Note: Bold values indicate the best performance.

**Table 3:** MSE and SSIM performance metrics across reconstruction methods.

	Method	SRNet w/o DSA	SRNet-P Only	SRNet w/o Illumination	SRNet
Metrics					
MSE		0.0032	0.0024	0.083	**0.0024**
SSIM		0.8413	0.8393	0.3442	**0.8724**

Note: Bold values indicate the best performance.

**Figure A1:**
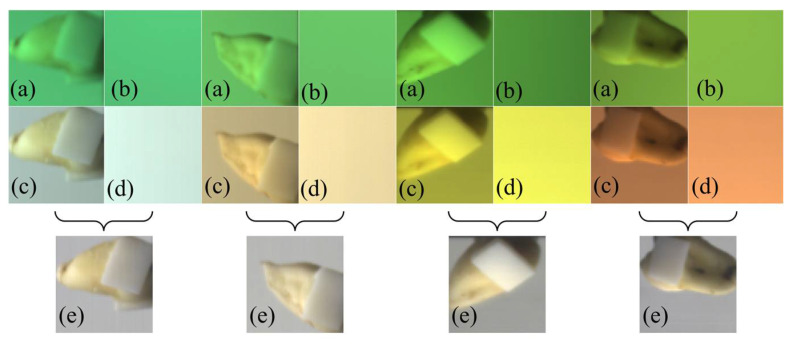
(**a**) tooth sample images acquired with a DSLR camera and subsequently processed, (**b**) white board images acquired with a DSLR camera and subsequently processed (illumination), (**c**) pseudo-color visualizations of tooth samples derived from hyperspectral images, (**d**) pseudo-color visualizations of white board derived from hyperspectral images (illumination), and (**e**) pseudo-color visualizations of tooth sample spectral reflectance images.

**Figure A2:**
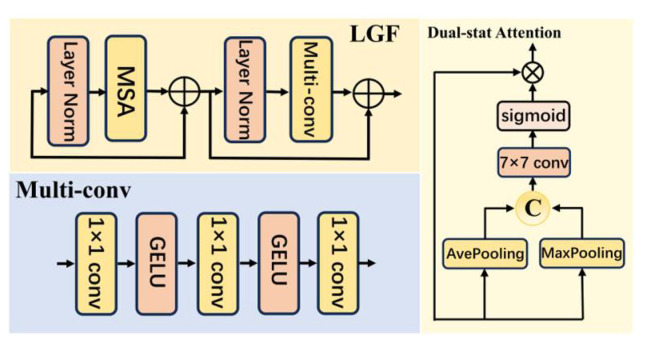
Detailed structure of LGF and DSA. The “C” in Figure A2 indicates that the two inputs are concatenated in the channel dimension. ⊕ indicates adding while ⊗ means multiplication.

**Figure A3:**
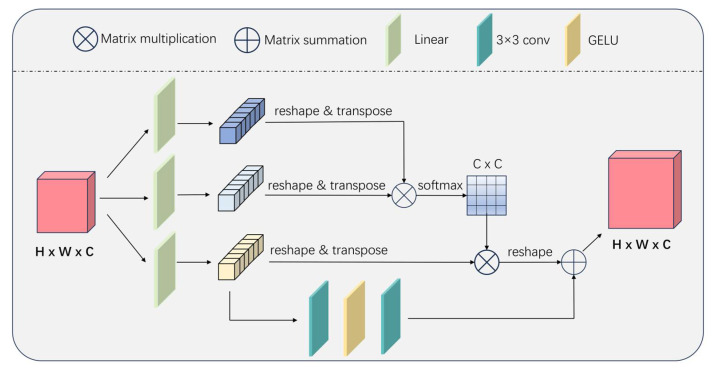
Details of the multi-head self-attention (MSA) block.

**Figure A4:**
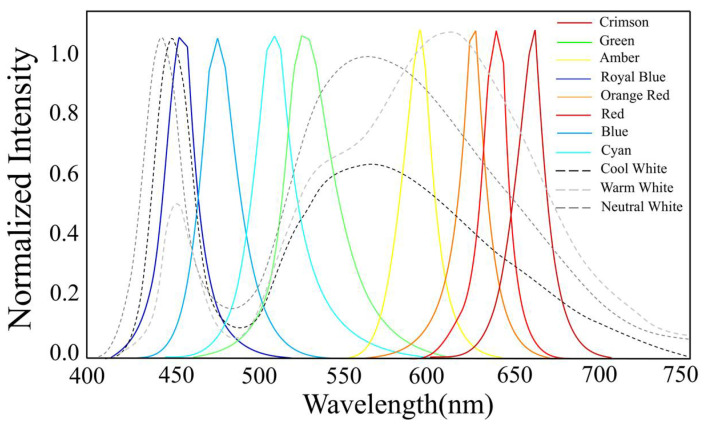
Normalized spectral intensity of diverse LED beads in the light box.

## Data Availability

The data are not publicly available due to privacy and ethical restrictions.

## Abbreviations

The following abbreviations are used in this manuscript:

IOS	Intraoral Scanner
HSI	Hyperspectral Image
LGF	Local–Global Fusion
DSA	Dual-Stat Attention
MSA	Multi-Head Self-Attention
SA	Spatial Attention
CA	Channel Attention
SSIM	Structural Similarity Index Measure
MSE	Mean Square Error
CCT	Correlated Color Temperature
DSLR	Digital Single-Lens Reflex

## Appendix A

This appendix presents a subset of the training samples utilized in this study. The samples in Figure A1 are selected to illustrate the diversity and characteristics of the dataset, including variations in spectral signatures and spatial features. These examples provide insight into the data used to train the proposed hyperspectral reconstruction model.

## Appendix B

Convolutional operations typically focus on local receptive fields, whereas attention mechanisms are capable of capturing long-range dependencies. By leveraging spatial and channel attention modules, the model can effectively capture global contextual information and enhance spatial–spectral feature representations. This capability is crucial for spectral imaging tasks, which demand precise characterization of features across diverse spatial positions and spectral bands.

Therefore, we proposed spatial–spectral enhancement modules, comprising local–global fusion (LGF) and dual-stat attention (DSA), which were integrated into SRNet to enhance performance in Step 1.

The local–global fusion (LGF) module addresses the challenge of simultaneously capturing fine-grained local spatial details and broad global contextual information in hyperspectral image reconstruction. Conventional convolutional networks often struggle to represent features across multiple scales, leading to incomplete spatial–spectral modeling and reduced reconstruction accuracy. By integrating features extracted at different receptive fields, the LGF module enhances the network’s ability to comprehensively capture complex spatial and spectral correlations inherent in hyperspectral data.

The dual-stat attention (DSA) module focuses on overcoming the redundancy and noise present in the numerous spectral channels of hyperspectral images, as well as the uneven importance of different spatial regions. Traditional convolutional operations lack the capability to dynamically recalibrate attention across channels and spatial locations, which may cause the network to distribute focus inefficiently. The DSA module adaptively computes attention weights based on statistical properties such as mean and standard deviation, allowing the network to emphasize informative spectral bands and spatial areas while suppressing irrelevant or noisy features. This selective feature enhancement improves both the robustness and accuracy of the spectral reflectance reconstruction.

### Appendix B.1. Local–Global Fusion (LGF)

As illustrated in Figure A2, the LGF module is composed of multiple submodules, including layer normalization, multi-head self-attention (MSA), and a multi-convolutional block. By incorporating residual connections, LGF effectively captures long-range dependencies while preserving the flow of information.

The network structure includes two primary components: the self-attention layer (MSA) for extracting global features and the feedforward network (multi-convolutional block) for enhancing the model’s nonlinear expressive capacity. The architecture of the multi-convolutional block is depicted in Figure A2.

Given an input feature map *F* ∈ *R^B×H×W×C^*, LGF consists of multiple sequential blocks, each performing multi-head self-attention (MSA) and multi-convolution (MC) operations with residual connections:

(A1) F←F+MSA(Z)

(A2) F←F+MC(Z)

#### Appendix B.1.1. Multi-Head Self-Attention

Given the input *X_in_* ∈ *R^H×W×C^* for MSA, it is first reshaped into a sequence of tokens. These tokens are subsequently transformed via linear projections into the query *Q* ∈ *R^HW×C^*, key *K* ∈ *R ^HW×C^*, and value *V* ∈ *R^HW×C^*, using the learnable weight matrices *W_Q_*, *W_K_*, *W_V_* ∈ *R^C×C^:*

(A3) $$Q=XW_{Q},K=XW_{K},V=XW_{V}$$

Subsequently, *Q*, *K*, and *V* are partitioned into N heads along the spectral channel dimension, resulting in *Q* = [*Q*_1_, …, *Q_N_*], *K* = [*K*_1_, …, *K_N_*], *V* = [*V*_1_, …, *V_N_*], where the dimension of each head is:

(A4) $$d_{h}=\frac{C}{N}$$

In MSA, each spectral representation is regarded as an individual token, and self-attention for the *head_j_* is computed as follows:

(A5) $$A_{j}=softmax(\sigma_{j}K_{j}^{T}Q_{j}),head_{j}=V_{j}A_{j}$$

where *K_j_^T^* indicates the transpose of matrix *K_j_*, and *σ_j_* ∈ *R* is a learnable parameter.

To mitigate spectral variations, a learnable parameter adjusts the matrix product *A_j_* within each head during the computation of *K_j_^T^Q_j_*.

Subsequently, the outputs from all *N* heads are concatenated and passed through a linear projection:

(A6) $$MSA(X)=(Concat_{j=1}^{N}(head_{j})W)+eps(V)$$

where *W* ∈ *R^C×C^* is a learnable weight matrix and *eps*(·) denotes the position embedding function.

Position embedding is critical for representing positional information across spectral channels, given that hyperspectral images are organized in ascending wavelength order along the spectral dimension. The detailed architecture of the Multi-Head Self-Attention (MSA) module is illustrated in Figure A3.

#### Appendix B.1.2. Multi-Convolution

The multi-convolution module is designed to enhance local feature extraction by applying a sequence of convolutions interleaved with nonlinear activations and separable convolution, efficiently capturing spatial details while reducing computational cost.

Given an input feature map *F* ∈ *R^B×^^C×^^H×^^W^*, the module first expands the channel dimension by a point-wise 1 × 1 convolution, then applies a nonlinear activation *ϕ*(·), followed by a 3 × 3 convolution, another nonlinear activation, and finally reduces the channel dimension back to C with another point-wise convolution. Formally, this can be expressed as:

(A7) $$Y=Conv_{1\times 1}(\phi (Conv_{3\times 3}(\phi (Conv_{1\times 1}(F)))))$$

where *ϕ*(·) denotes GELU activation.

By reducing parameters and computational cost compared to standard convolutions, it efficiently captures local spatial correlations, while nonlinear activations enable the modeling of more complex feature interactions.

### Appendix B.2. Dual-Stat Attention

The dual-stat attention (DSA) module is designed to enhance spatial feature representation by leveraging statistical pooling operations. Specifically, it utilizes both average pooling and max pooling along the channel dimension to extract complementary spatial descriptors from the input feature map. Average pooling captures the global contextual distribution of features, while max pooling emphasizes localized salient responses.

Also, given an input feature map *F* ∈ *R^B×^^C×^^H×^^W^*, DSA computes the average-pooled map and max-pooled map across the channel dimension as:

(A8) $$M_{avg}(i,j)=\frac{1}{C}\sum_{c=1}^{C}F_{c,i,j},M_{max}(i,j)=\underset{1\leq c\leq C}{\max}F_{c,i,j}$$

These two maps are concatenated along the channel axis and passed through a convolutional layer followed by a sigmoid activation to generate a spatial attention map *A_S_* ∈ *R^B×^*^1*×*^*^H×^^W^*:

(A9) $$A_{s}=\sigma (Conv_{7\times 7}(Concat(M_{avg},M_{max})))$$

where *Concat*(*A, B*) denotes concatenation and *σ*(*·*) indicates the sigmoid function.

The output feature map is then obtained by element-wise multiplication of the input and the spatial attention map broadcasted over channels:

(A10) $$Y=F⊗A_{s}$$

By combining these two statistical views, DSA generates a more informative and balanced spatial attention map. This attention is then used to selectively reweight spatial locations, enabling the network to focus on regions that are both contextually important and structurally distinctive. The DSA module is lightweight, fully convolutional, and integrates seamlessly within the encoder–decoder framework, particularly along skip connections to enhance multi-scale feature fusion.

## Appendix C

Table A1 summarizes the key statistical characteristics of the dataset used in this study, including the distribution of light-source types, the number of samples acquired under each illumination condition, and other essential acquisition parameters. This statistical overview provides a concise yet comprehensive characterization of the dataset, supporting the interpretation of subsequent experimental results.

**Table A1:** Dataset acquisition specifications.

Category	Parameter	Value
Light Source	Number of Type	16
	Type	Please refer to Section 3.2
	Intensity	Please refer to Appendix D for details.
Light Source	Spectral Range	380–780 nm
Tooth samples	Ceramic Thickness	0.4–2 mm
	Ceramic Material	White zirconia presintered discs composed of ZrO_2_ + HfO_2_ + Y_2_O_3_ (total content > 99%) with Al_2_O_3_ content less than 0.5% and yttria (Y_2_O_3_) doping at 3 mol%.
	Number of Teeth	10
	Source of teeth	Extracted teeth from West China Hospital, Sichuan University, Dec 2023, due to periodontitis and orthodontics.
Camera (RGB)	Model	Nikon D3X
	Resolution	6048 × 4032 pixels
Camera (Spectral)	Model	Specim-IQ
	Spectral range	397.32–1003.58 nm
Viewpoint variation	Measured angle	Simulated by augmentation
Dataset split	Train/Val/Test	3000/300/700.

## Appendix D

The lighting module of the experimental light box comprised eleven different types of light-emitting diodes (LEDs), including three variants of white light and eight monochromatic sources. The detailed characteristics of these LEDs are summarized in Table A2. A total of 1700 LEDs were systematically arranged on a 700 mm × 400 mm circuit board array. Through precise modulation of the individual LED output intensities, a visible illumination spectrum spanning the wavelength range of 380–780 nm was successfully generated.

**Table A2:** Models, parameters, and quantities of the LED beads in the light box. Abbreviations: Std. λ: standard peak wavelength; Meas. λ: measured peak wavelength; Min. Φ: minimum luminous flux; FWHM: full width at half maximum.

Color	Model	Std. λ (nm/K)	Meas. λ (nm/K)	Min. Φ (lm)	FWHM (nm)	Quantity
Royal Blue	LXM2-PR02-0100	445–450	447	100	24	160
Blue	LXM2-PB01-0030	475–480	479	30	33	160
Cyan	LXML-PE01-0070	505–510	506	70	30	160
Green	LXM2-PM01-0090	520–525	522	90	30	160
Amber	LXML-PL01-0030	592–594	593	30	28	160
Orange-Red	LXM3-PH01-0070	610–620	615	70	28	160
Red	LXM2-PD01-0040	620–630	626	40	29	160
Deep Red	LXM2-PD01-0030	650–660	657	30	29	160
Warm White	LXML-PW30	3000 K	3000 K	30	N/A	140
Neutral White	LXML-PWN2	4100 K	4000 K	130	N/A	140
Cool White	LXML-PWC2	5650 K	5582 K	80	N/A	140

The normalized emission spectra of the various LED types are illustrated in Figure A4. To ensure homogenous and uniform illumination, an optical diffuser was positioned within the optical path. This diffuser functions to spatially scatter the light, thereby mitigating sharp luminance gradients and preventing uneven brightness distribution caused by discrete LED sources.

## Appendix E

Table A3 summarizes the computational requirements of the SRNet components, including the number of parameters (Params), inference time per image, and peak memory consumption. The peak memory consumption was measured on an NVIDIA RTX 5070 GPU with a batch size of 4, using input images of size 64 × 64 × 3. As shown, SRNet-P, which has the largest number of parameters, requires the longest inference time, while SRNet-L achieves faster inference due to its reduced complexity. The SRNet-L for fusion demonstrates a slight increase in computational cost compared to SRNet-L alone, reflecting the additional processing for feature fusion. This information provides readers with a clear understanding of the trade-offs between model complexity and runtime performance, which is crucial for practical deployment in clinical or embedded systems.

**Table A3:** Model complexity and computational requirements of SRNet components, including number of parameters, inference time, and peak memory consumption.

Model	Params (M)	Inference Time (ms)	Peak Memory Consumption	Hardware Environment
SRNet-P	4.594	23.34	4.5 GB	
SRNet-L	4.101	15.23		Nvidia RTX 5070
SRNet-L (fusion)	4.136	16.56		
